# Metabolic Reprogramming: Strategy for Ischemic Stroke Treatment by Ischemic Preconditioning

**DOI:** 10.3390/biology10050424

**Published:** 2021-05-11

**Authors:** Jing Liang, Rongrong Han, Bing Zhou

**Affiliations:** 1 Beijing Advanced Innovation Center for Big Data-Based Precision Medicine, Interdisciplinary Innovation Institute of Medicine and Engineering, Beihang University, Beijing 100191, China; liangjing2019@buaa.edu.cn (J.L.); hanrongrong2019@buaa.edu.cn (R.H.); 2 School of Engineering Medicine, Beihang University, Beijing 100191, China

**Keywords:** ischemic stroke, ischemic preconditioning (IPC), metabolic reprogramming

## Abstract

**Simple Summary:**

Ischemic preconditioning triggers endogenous neuroprotection to defend against subsequent, more severe cerebral ischemia. However, the underlying biological mechanisms of ischemic preconditioning are still confusing. Here we provide a summary of metabolic mechanism of ischemic preconditioning when it is applied to ischemic stroke. It has been found that metabolic disorder is a determinant of the incidence and progression of stroke. To maintain the cerebral activity transiently, upon ischemia onset, brain tissues enhance their metabolic plasticity, mainly through energy metabolic reprogramming and antioxidant defense. Growing evidence suggests that ischemic preconditioning takes advantage of brain plasticity for its neuroprotective purposes, among which, metabolic reprogramming is crucial to co-ordinate the metabolic imbalance, especially for energy and redox homeostasis. Objectively, the study on metabolic reprogramming of ischemic preconditioning is still in its infancy, such as, there are extremely few studies on the spatiotemporal variation, aging influence, and astrocyte-neuron interactions in metabolic reprogramming of ischemic preconditioning. Further study focus on ischemic preconditioning metabolic reprogramming is needed, and it will be valuable for exploring the mechanisms of ischemic preconditioning, and will be greatly beneficial for the understanding of ischemic stroke treatment and standardized application of ischemic preconditioning.

**Abstract:**

Stroke is one of the leading causes of death and permanent disability worldwide. Ischemic preconditioning (IPC) is an endogenous protective strategy, which has been reported to exhibit a significant neuroprotective effect in reducing the incidence of ischemic stroke. However, the underlying neuroprotective mechanisms of IPC remain elusive. An increased understanding of the pathogenic mechanisms of stroke and IPC serves to highlight the importance of metabolic reprogramming. In this review, we summarize the metabolic disorder and metabolic plasticity in the incidence and progression of ischemic stroke. We also elaborate how IPC fully mobilizes the metabolic reprogramming to maintain brain metabolic homeostasis, especially for energy and redox homeostasis, and finally protects brain function in the event of an ischemic stroke.

## 1. Introduction

**Figure 1:**
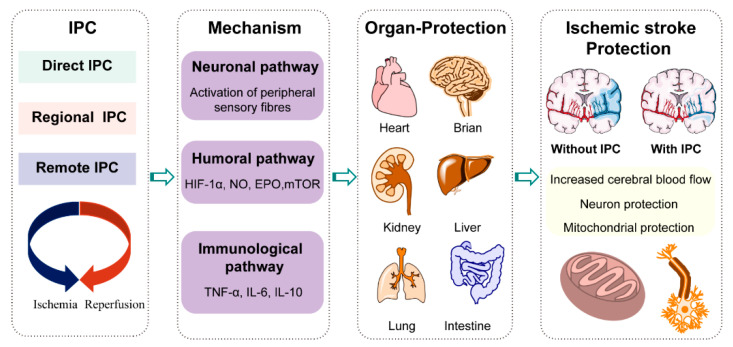
General description of ischemic preconditioning (IPC), in which several cycles of brief non-lethal ischemia and reperfusion are applied either directly, regionally, or remotely. Through neuronal, humoral, and immunological pathways, IPC confers protection against subsequent, more severe, and lethal ischemia. The ischemic protection of IPC has been applied in various organs, such as the heart, brain, kidney, liver, lungs, and intestine. For ischemic stroke, IPC can reduce the infarct size and improve prognosis, which is supported by increasing the cerebral blood flow (CBF), protecting mitochondrial function, and maintaining neuronal activity.

Stroke is a leading cause of death and permanent disability, imposing heavy social and family burdens [1,2]. Ischemic stroke is typically caused by blood vessel blockage, which accounts for approximately 87% of all stroke cases. Over the past few decades, considerable progress has been made in ischemic stroke treatment, typically in intravenous thrombolysis and mechanical thrombectomy. However, these conventional therapies have a narrow therapeutic window: the effective intravenous thrombolytic therapy is within 4.5 h of onset, and that of intra-arterial thrombectomy is within 6 h of onset [3], resulting in only a minority (3–5%) of stroke patients being able to receive these therapies [4]. Meanwhile, although restoring blood flow by thrombolysis and thrombectomy is essential in limiting ischemic neuronal damage, substantial neuronal, glial, and neurovascular damages may still occur, particularly due to reperfusion injury of the penumbra [5]. Furthermore, there exist some serious contraindications and complications; for example, thrombolytic agents have been associated with symptomatic intracerebral hemorrhage [6]. Thus, there is an urgent need to develop new treatment strategies for ischemic stroke. A field of research that continues to show promise in developing therapies for ischemic stroke is ischemic preconditioning (IPC). IPC is an endogenous metabolic protective strategy, whereby several cycles of brief, non-lethal ischemia, followed by reperfusion, confer protection against subsequent, more severe, and lethal ischemia. It was first discovered by Murry et al., in the canine heart, in 1986 [7]. To date, IPC has been replicated in humans and other species, and applied to other organs and tissues (e.g., the brain and kidney). Direct IPC is conducted by brief, direct, repetitive clamping of the target artery, while regional IPC involves a repetitive occlusion of the circumflex artery, which is near to the target artery. In remote IPC, inflation of a blood-pressure cuff on the arm or leg is used [8]. IPC has been reported to exhibit a significant neuroprotective effect, remarkably reducing the incidence of ischemic stroke and improving the prognosis in patients with stroke [9]. As IPC is innocuous, cost-effective, and has fewer or no contraindications, and has exciting new prospects in the broader management of ischemic stroke (Figure 1).

However, the underlying neuroprotection mechanisms of IPC remain elusive. Increasing understanding of the related pathogenic mechanisms serves to highlight the importance of metabolic regulation. It has been found that metabolic disorders are a determinant of the incidence and progression of stroke. Moreover, the brain utilizes metabolic plasticity, a protective response to stroke injury. Elucidation of these endogenous defense mechanisms against ischemic injury is considered crucial for the development of novel stroke therapies. Increasing evidence has shown that IPC takes advantage of brain plasticity and endogenous defense mechanisms for its neuroprotective purposes, among which metabolic reprogramming is crucial to co-ordinate the metabolic imbalance; support demands for body energy, biomass, redox maintenance, and cellular communication; and, finally, affecting pathophysiological alterations in ischemic stroke. Nevertheless, metabolic reprogramming is a relatively new area for understanding the mechanisms of IPC, to the best of our knowledge, no relevant review has yet been published. Therefore, the provision of a summary on the progress of the metabolic regulation in ischemic stroke and IPC will serve to provide new ideas for ischemic stroke therapies.

### 1.1. Metabolic Disorder and Metabolic Plasticity in Ischemic Stroke: Key Considerations and Major Features

**Figure 2:**
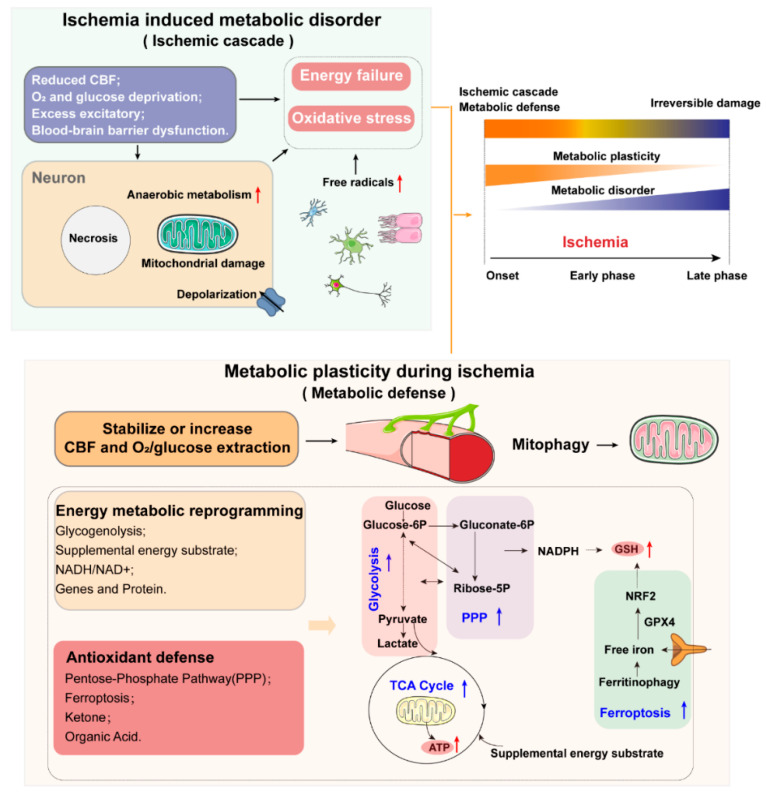
Metabolic disorder and metabolic plasticity in ischemic stroke: Upon ischemia onset, a sharp reduction of regional CBF results in oxygen and glucose deprivation, followed by excess excitatory and blood–brain barrier dysfunction. Neurons experience mitochondrial dysfunction, shifting the cellular machinery from aerobic to anaerobic metabolism, and a decrease of ATP production, directly resulting in energy failure. In the meantime, free radicals trigger oxidative stress, which further induce damage to nucleic acid bases, lipids, and proteins, ultimately leading to cell death by necrosis or apoptosis. To defend against this ischemic cascade, upon ischemia onset, brain tissues enhance their metabolic plasticity, in order to maintain the cerebral activity transiently, mainly through the regulation of CBF, extraction of oxygen and glucose, energy metabolic reprogramming, antioxidant defense, and mitophagy. However, with persistent ischemia, irreversible damage may occur in the affected brain areas.

Recent research has shown that metabolic disorders have significant effects, both before and after the onset of ischemic stroke. Long-term metabolic disorders, such as metabolic syndrome (MetS), increase the probability of occurrence of ischemic stroke. The most immediate biochemical alterations in neurons affected by ischemia are mitochondrial dysfunction, shifting the cellular machinery from aerobic to anaerobic metabolism, and energy production decreasing from 32 adenosine triphosphate (ATP) molecules to 2 ATP molecules. Energy failure leads to the depolarization of neurons and activation of specific glutamate receptors dramatically, which further induce the failure of the transmembrane electrochemical gradient established by the Na+, K+-ATPase pump. Then, the accumulated free radicals damage cell membranes, mitochondria, and DNA, thus triggering caspase-mediated cell death. To defend against this ischemic cascade, upon the onset of ischemia, brain tissues enhance their metabolic plasticity to maintain the cerebral activity transiently, mainly through the regulation of cerebral blood flow (CBF), mitochondrial adaption, and other defense systems; however, with persistent ischemia, irreversible damage can occur in the affected brain areas. It should be noted that metabolic reprogramming is a double-edged sword: the overactivation of metabolic reprogramming under ischemia may lead to secondary brain damage (Figure 2).

### 1.2. Cerebral Blood Flow

Ischemic stroke is the consequence of a sharp reduction of regional cerebral blood flow (CBF), resulting in oxygen and glucose deprivation (OGD). There are two major affected zones in an ischemic brain: The infarct core, surrounded by an ischemic penumbra. The normal CBF in “healthy normal men” is 54 mL/100 g per minute. It has been noted that a minimal CBF of 18 mL/100 g per minute is needed to maintain normal electroencephalographic (EEG) activity. In the ischemic penumbra, a further decrease in CBF leads to neuronal electrical silence and a synaptic activity decrease to preserve energy stores, while energy metabolism is partially preserved to transiently sustain tissue viability. When the CBF is below 10 mL/100 g per minute, irreversible cellular injury will occur, and the infarct core forms [10]. Timely interventions are effective for avoiding the progression of the penumbra into infarction. In response to ischemia, there exists a cerebral vessel autoregulatory mechanism, inducing the enhancement of cerebral collateral circulation and vasodilation, in order to stabilize or increase the CBF and oxygen/glucose extraction for viable neurons. The cerebral collateral circulation—known as the subsidiary network of vascular channels—can stabilize the CBF when principal conduits fail. Collaterals are demonstrated to be strong predictors of both response to endovascular therapy and functional outcomes [11]. In addition, recent findings have indicated that mitochondria may represent a useful target to restore CBF after stroke, as it has been shown that ATP, adenosine monophosphate (AMP), and adenosine diphosphate (ADP) can alter cerebrovascular tone via plasmalemmal purinergic receptors [12]. Jaggar et al. showed that the depolarization of mitochondria by diazoxide promoted the relaxation of vascular smooth muscle (VSM) cells in endothelium-denuded cerebral arteries or freshly dissociated VSM, through the generation and localized effects of reactive oxygen species (ROS) [13]. The relaxation of VSM can also be indirectly regulated by the action of NO and other vasoactive agents. Therefore, it can be seen that mitochondrial mechanism is an important, but underutilized, target for improving CBF and decreasing brain injury in stroke patients [14].

### 1.3. Mitochondria and Energy Metabolic Reprogramming

#### 1.3.1. Mitochondria and Energy Substrate and Supply

Mitochondria are signaling, bioenergetic, and biosynthetic organelles. These multifaceted functions make them important cellular stress sensors, and they drive metabolic reprogramming for cellular adaptation to harsh environments, such as nutrient depletion or hypoxia [15]. Upon the onset of ischemia, the level of AMP dramatically decreases in the cortex and hippocampus tissues, indicating energy failure in these tissues [16]. To defend against this, the brain shifts the cellular machinery from aerobic to anaerobic metabolism. The accumulation of glucose and glycolytic intermediates is a prominent feature of brain ischemia-induced metabolic disturbance in rodents. Lactate in cerebrospinal fluid (CSF) has also been found to be higher in stroke patients [17]. More glycolytic intermediates divert into the pentose–phosphate pathway (PPP), while the entrance of pyruvate for mitochondrial oxidation is downregulated [18].

At the onset of ischemic stroke, in order to maintain the energy demand, compensatory pathways are initiated, comprising a major metabolic reprogramming strategy including glycogen metabolism, lactate metabolism, amino acid metabolism, and lipid metabolism. ➀ Glycogen: The metabolism of glycogen is critical for the release of stored glucose. Wender et al. found that, after 60 min of glucose deprivation, astrocytes in the rat optic nerve (a CNS white matter tract) drove glycogen to be broken down to lactate, which was then transferred to fuel axons [19]. Consistent with this data, strategies aimed at increasing astrocyte glycogen have been successfully applied for mitigating neuronal loss [20]. ➁ Lactate: Studies in the past two decades have indicated that lactate is also an efficient energy substrate for maintaining neuronal integrity and functioning during cerebral ischemia [21]. Direct intracerebroventricular or intravenous administration of lactate protected mouse brains against ischemic injury [22]. Meanwhile, exogenous supplementation of lactate has shown remarkable effects in traumatic brain injury therapy [23]. ➂ Amino acids: To sustain fuel oxidation by the tricarboxylic acid (TCA) cycle, nerve cells upregulate glutaminolysis and use of fatty acids and branched chain amino acids. Glutamine is the most abundant free amino acid in human blood, which is converted to glutamate in mitochondria by glutaminase (GLS). Glutamate can be converted back to α-ketoglutarate by oxidative deamination in astrocytes, to undergo further oxidation in the TCA cycle for the purpose of energy generation [24]. Research has found that L-glutamine reduced brain infarct volume and promoted neurobehavioral recovery in cerebral ischemic mice [25]. Other significant changes in amino acid metabolic pathways have also been confirmed upon ischemia: the levels of some well-known energy-providing amino acids, such as leucine, isoleucine, valine, tyrosine, and lysine, increased significantly in brain tissues of mice treated by IPC, indicating that proteolysis was up-regulated [16]. ➃ Ketones: Growing evidence has indicated that ketone bodies are beneficial in treating stroke [26], mainly β-hydroxybutyrate (β-HB) and acetoacetate, which can substitute for glucose under conditions of energy deficiency in the brain for cellular fuel [27]. A previous study has demonstrated that cerebral ischemia caused a ketogenic response, shown through the enhancement of hepatic free fatty acids β-oxidation and increasement of serum and brain β-hydroxybutyrate levels [28]. Most ketones are generated in the liver, while the transport of ketone bodies across the blood-brain barrier (BBB) is the limiting step. In the brain, astrocytes can also generate ketone bodies from fatty acid β-oxidation. All brain cell types are able to uptake ketones; the ketones are then metabolized to acetyl-CoA to support the cell energy [29]. ➄ Reduced nicotinamide adenine dinucleotide(NADH)/nicotinamide adenine dinucleotide (NAD^+^): The NAD^+^ and NADH redox couple is essential for a variety of electron exchange-dependent biochemical reactions, and serves as a cofactor for enzymes involved in glycolysis, the oxidative decarboxylation of pyruvate to acetyl-CoA, fatty acid β-oxidation, and TCA cycle. During glycolysis, NAD^+^ is reduced to NADH, which is then oxidized by complex I of the mitochondrial electron transport chain (ETC) to supply the necessary proton gradient for ATP production. NAD^+^ levels and the NAD^+^/NADH redox couple provide a readout and regulator for cellular energy metabolism [30]. Changes in the cerebral NAD^+^ pool under ischemia have been studied in detail. At the onset of ischemia, NAD^+^ levels decrease within 30 min, a second depletion occurs at 6 h of reperfusion (when necrosis is prominent), and a third depletion of NAD^+^ happens at 24 h (when apoptosis is prominent). Finally, the NAD^+^ pool declines by approximately 35–50%. The malate–aspartate shuttle (MAS) is considered the most important NAD^+^/NADH shuttle in neurons, playing a prominent role in neuronal mitochondrial respiration. The MAS has been implicated as potentially dysregulated during cerebral ischemia [31]. Rapid NAD^+^ depletion inevitably disrupts intracellular energy homeostasis. Direct NAD^+^ repletion, either in animal or in cultured neurons, markedly reduced ischemic cell death and DNA damage [32,33]. In response to the NAD^+^ decline, NAMPT was upregulated in brain, plasma, and cultured neurons, which is the rate-limiting enzyme in mammalian NAD^+^ salvage biosynthesis [34]. Increased pools of NAMPT and NAD^+^ are protective against oxygen–glucose deprivation, as well as playing a crucial role in cell energy maintenance.

#### 1.3.2. Genes and Protein Related with Energy Metabolism

Metabolic reprogramming during ischemic stroke is also reflected in the large changes of genes and proteins related to carbon and lipid metabolism. Betti et al. investigated genomic DNA from 501 ischemic stroke patients and 1211 comparable controls, and identified significant genetic associations between premature ischemic stroke in BHMT, CBS, FOLH1, MTR, PON2, TCN2, and TYMS genes, which are involved in methionine metabolism [35]. Other metabolic-related genes in the pathogenesis of ischemic stroke include MTHFR, CBS, and MTR, which are involved in homocysteine metabolism, and apo E, LPL, CETP, ABCA1, apo AI, apo CIII, apo AIV, apo AV, apo B, apo H, apo(a), PON1/2/3, and LDLR/LOX-1, which are involved in lipid metabolism [36]. At the protein level, glutamate oxaloacetate transaminase (GOT), which can metabolize glutamate into TCA intermediates, is induced during acute ischemic stroke (AIS), and may therefore be useful to harness excess neurotoxic extracellular glutamate during AIS [37]. Raf kinase inhibitory protein (RKIP) is involved in the protective effect against stroke: Li et al. revealed that RKIP overexpression markedly reduced the necrotic area after ischemic stroke, mainly reflected in the metabolism of energy, amino acids, and lipids [38].

### 1.4. Oxidative Stress and Antioxidant Defense

#### 1.4.1. Oxidative Stress

Reactive oxygen species (ROS), in the form of superoxide and hydroxyl free radicals, as well as hydrogen peroxide, are produced from multiple physiological reactions, including electron transport by the ETC and nicotinamide adenine dinucleotide phosphate (NADPH) oxidases, which are often exacerbated under hypoxic micro-environments. Furthermore, the accumulation of the TCA intermediate succinate is also responsible for mitochondrial ROS production during ischemic reperfusion [39]. Mitochondria are major contributors to cellular ROS, and there are multiple antioxidant pathways to neutralize ROS, including superoxide dismutase (SOD2), glutathione, thioredoxin, and peroxiredoxins. When ischemic stroke occurs, a rapid increase in the production of ROS rapidly overwhelms the antioxidant defenses, which are inadequate to completely clear the ROS. Under these circumstances, oxidative stress occurs, which further induces damage to nucleic acid bases, lipids, and proteins, ultimately leading to cell death by necrosis or apoptosis [40].

#### 1.4.2. Antioxidant Defense

To defend against oxidative stress, cells have developed complex systems that exploit and defend against this dilemma. These include: ➀ NADP^+^/NADPH. NADP^+^ and its reduced counterpart, NADPH, are mainly required for anabolic reactions and cellular oxidative-stress defense. NADP^+^ is an essential cofactor for the rate-limiting step of the pentose–phosphate pathway (PPP). This pathway can produce precursors to synthesize nucleotides and aromatic amino acids, generating cytosolic NADPH simultaneously [30]. Once ischemic stroke occurs, the PPP is boosted and more glycolytic intermediates are diverted into the PPP to sustain NADPH production [18]. It has been found that direct administration of NADPH can significantly reduce infarct volume, improving post-stroke survival and neurological function recovery in mouse and rat stroke models, with a remarkable increase in the level of the reduced form of glutathione (GSH), while decreasing ROS levels [41]. ➁ Ferroptosis. During ischemia, the depletion of GSH and NADPH causes an iron-dependent accumulation of lipid hydroperoxides to lethal levels, thus inducing cell death, which is defined as ferroptosis [42]. The biochemical control of ferroptosis includes amino acid metabolism, glutathione metabolism, lipid metabolism, iron metabolism, and other metabolic pathways [43]. First, the availability of cysteine is the limiting link in GSH biosynthesis. Glutamate can be exchanged for cystine in a 1:1 ratio, such that the accumulation of extracellular glutamate could trigger ferroptosis in physiological contexts [44]. In the absence of glutamine (or when glutaminolysis is inhibited), cystine starvation and ferroptosis occur. Second, polyunsaturated fatty acids are susceptible to lipid peroxidation and are necessary for ferroptosis [45]. Hence, the abundance and localization of polyunsaturated fatty acids are crucial for the degree of lipid peroxidation that occurs in cells. Third, iron deficiency has been associated with an increased risk of ischemic stroke [46]. Iron is essential for the accumulation of lipid peroxides and execution of ferroptosis. Within cells, the selective autophagy of ferritin (abbreviated as ferritinophagy), by modulating iron metabolism and controlling iron availability, occurs to enhance ferroptosis sensitivity [47]. Studies have revealed that inhibitors of ferroptosis, such as ferrostatins, carvacrol, and liproxstatins, could protect against cerebral ischemic injury in rodent models [43,48]. ➂ Ketone. A previous study has shown ketone bodies to reduce ROS by using NADH as an electron donor. Yin, J. et al. found that ketone treatment in mice at 30 min after ischemia enhanced mitochondrial function, reduced oxidative stress and, therefore, reduced infarct volume [49]. ➃ Organic Acids. Zhang et al. revealed that, upon ischemia, the levels of three oxidative stress-related metabolites—succinate, taurine, and malonate—were dramatically disturbed in the cortex tissues of ischemic mice: taurine decreased, while malonate and succinate increased [16]. Thus, the pretreatment of cells with taurine could reduce oxidative stress [50]. We can see that antioxidant defense plays an important role in the redox control, which may promote new therapeutic strategies for ischemic stroke in the future.

### 1.5. Mitochondria and Mitophagy

The adult brain occupies less than 2% of the body’s weight, yet it consumes 25% of the cardiac output at rest and accounts for 20% of the total energy production of the body. The major function of mitochondria is ATP production, but they perform many other roles as well, including biosynthetic metabolism, generation of ROS, redox molecules and metabolites, and regulation of cell signaling and cell death. Mitochondria lie at the key location for neuronal survival [51]. It has been demonstrated that mitochondria are a major target in ischemic injury. After hypoxic-ischemic insult, the perturbation of mitochondrial homeostasis can profoundly alter the ATP production and intracellular cellular energy status, leading to apoptotic cell death in the presence of increased ROS production, calcium accumulation, opening of mitochondrial permeability transition pores (mPTPs), and releasing cytochrome C [52,53]. Therefore, a fastidious quality control system is important: as is well-known, mitochondrial dysfunction can initiate mitochondrial autophagy, which was first named “mitophagy” by Lemasters [54]. The clearance of damaged mitochondria through mitophagy is critical for cellular fitness, as dysfunctional mitochondria can impair ETC function and increase oxidative stress. Mitophagy is also essential in sustaining mitochondrial homeostasis, biogenesis, and the total number and quality of mitochondria. It has recently been demonstrated that mitophagy is highly involved in ischemic stroke and could be neuroprotective; furthermore, insufficient or altered mitophagy can lead to cell death and may promote the development and propagation of neurodegeneration [55,56]. Thus, we propose that mitophagy could be developed as an effective and potential target for the treatment of ischemic stroke.

### 1.6. Metabolic Syndrome and Ischemic Stroke

Metabolic syndrome (MetS) is a common metabolic disorder, involving a constellation of insulin resistance, abdominal obesity, hypertension, and dyslipidemia. An increasing number of studies have shown that epidemiologic changes are likely responsible for the observed rise of stroke incidence (Table 1). The pathophysiology of MetS seems to be largely attributable to the metabolic disorder caused by insulin resistance, with glucose intolerance and excessive flux of fatty acids also being implicated [57]. It will be a further explanation that the pathophysiological mechanisms in ischemic stroke are closely related to metabolic disorder.

Overall, metabolic reprogramming is a stress-protectant mechanism for brain tissues under ischemia, which can sustain cerebral cell survival in specified period, but will be invalidated if no effective interventions to recover glucose and oxygen supply are implemented for a prolonged stage. Furthermore, metabolic reprogramming is a double-edged sword; for example, the enhancement of glucose uptake and glycolysis can provide ATP faster, but the ongoing delivery of large amounts of glucose to the ischemic tissue along with an anaerobic glycolysis shift can adversely promote lactic acidosis, thus leading to tissue necrosis. Therefore, how to accurately and effectively utilize the metabolic reprogramming strategy is crucial, with which we anticipate its broad application in the prevention and treatment of ischemic stroke.

## 2. Metabolic Reprogramming in Ischemic Stroke Treatment by Ischemic Preconditioning

Metabolic disorder and metabolic plasticity are salient features triggered by ischemia. Metabolic reprogramming to maintain metabolic homeostasis, by correcting the metabolic disorder and enhancing metabolic plasticity, serves as an attractive potential therapeutic strategy for ischemic stroke. IPC is neuroprotective for ischemic stroke, but the precise mechanisms through which it exerts protection against ischemic insults remain unclear at present. Excitingly, emerging evidence from recent research has indicated that metabolic reprogramming may be the crucial neuroprotective mechanism of IPC for ischemia treatment.

### 2.1. Role of Metabolic Reprogramming in Metabolic Homeostasis

The brain is an unusual organ, having the highest metabolic activity and energy requirement by mass. This necessitates that the brain has reliable mechanisms to adequately protect its metabolic homeostasis. In steady-state, cells sustain themselves catabolically by using glycolytic carbons to fuel the TCA cycle. TCA cycle reactions yield metabolite intermediates and energetic precursors for oxidative phosphorylation. However, in response to changes in the micro-environment, metabolic reprogramming is notably crucial to maintaining metabolic homeostasis. As we showed in Section 1.2 and Section 1.3, under oxygen and glucose deprivation (OGD), the brain experiences a shift of the cerebral metabolism from glucose pathways to compensatory pathways, taking energy from other metabolic substrates, such as ketones, amino acids, endogenous carbohydrates, and lactate, in order to sustain energy and redox homeostasis.

**Figure 3:**
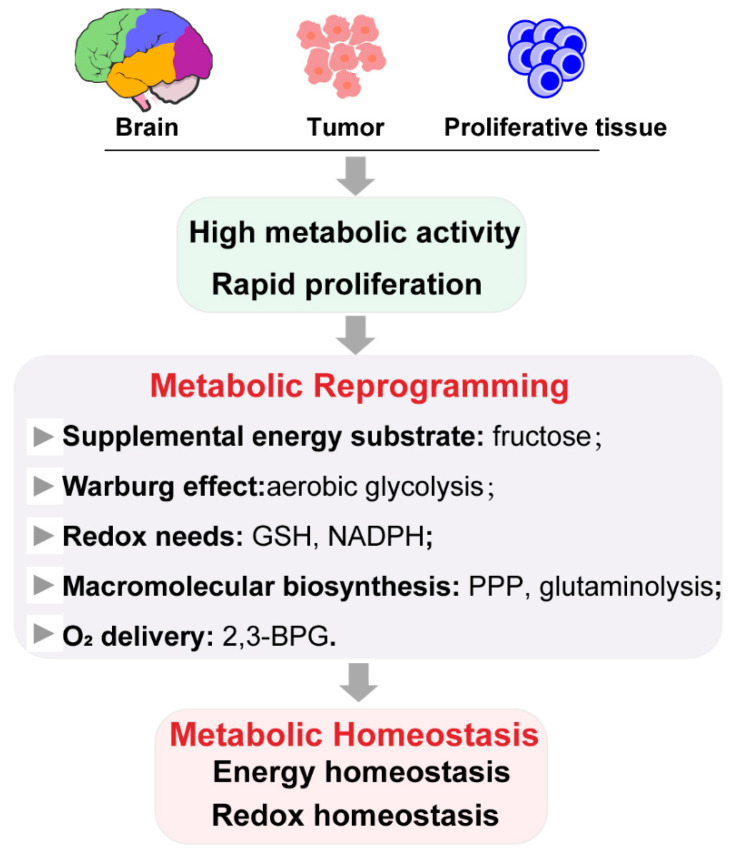
Metabolic reprogramming for metabolic homeostasis maintenance. Brain, tumor, and proliferative tissues have high metabolic activity and energy requirements, necessitating that they have reliable mechanisms to adequately protect their metabolic homeostasis. Metabolic reprogramming is notably crucial in this regard, especially for energy and redox homeostasis maintenance.

However, research on metabolic reprogramming in the neuroscience field is still in its infancy. Expanding fascinating horizons in metabolism of other cells under hypoxia or hypoglycemia may promote new inspirations. It is common that tumor cells reside in nutrient- and oxygen-poor environments, such that they adapt, through multiple metabolic reprogramming, to meet the energy, macromolecular biosynthesis, and redox needs required for rapid proliferation [63]. The most famous metabolic reprogramming process is the Warburg effect: Switching the energy metabolism largely to glycolysis, even in the presence of oxygen, implicating an increased rate of glucose uptake by cancer cells. This enhanced glycolysis drives the generation of energy-rich molecules (e.g., ATP, NADH, and NADPH) and the supply of carbon pool for the synthesis of amino acids, nucleotides, and lipids [64]. Fructose can be readily catabolized to fuel fatty acid synthesis and palmitoleic acid generation by lung cancer cells, as a glucose alternative [65]. Up-regulation of the PPP is frequently observed in tumors, in order to increase the production of NADPH and ribulose-5-phosphate, promote glutathione production, and increase nucleic acid and fatty acid synthesis, helping cells to counteract oxidative stress and facilitate DNA damage repairs. In biosynthetic pathways, cancer cells require that intermediate pools are maintained. Two activities that provide compensatory fluxes to “refill” the TCA cycle, respectively, are glutaminolysis (which produces α-ketoglutarate from glutamine) and oxidation of the branched-chain amino acids and fatty acids [66,67]. Immune cells also have distinct metabolic programs, in order to meet the energetic and biosynthetic requirements of their ever-changing micro-environments. Numerous in vitro, in vivo, and clinical studies have indicated that influenza infection induces hyperglycolysis in infected cells, activated immune cells, foci, and lymph nodes [68]. α-ketoglutarate, produced by glutaminolysis, is vital for alternatively M2-activated macrophages [69]. Furthermore, acute-on-chronic liver failure (ACLF) induces hyperammonemia and hypoxia in hepatocytes. The metabolic characteristics of HBV-related ACLF patients revealed the inhibition of glycolysis, TCA and urea cycle, and the enhancement of fatty acid oxidation and glutamine anaplerosis [70]. Under high altitude or chronic kidney disease, hypoxia-responsive sphingosine-1-phosphate (S1P) promotes erythrocyte glycolysis, channeling glucose metabolism toward Rapoport–Luebering Shunt and inducing 2,3-bisphosphoglycerate (2,3-BPG) production for O_2_ delivery [71,72]. Such emerging evidence of the metabolic reprogramming involved in metabolic homeostasis on the progression of different diseases has revealed that metabolic reprogramming is an important stress-protective mechanism, which plays a key role in many biological activities. The pathway mainly involves glycolysis, TCA cycle, PPP, and glutaminolysis to maintain the energy and redox homeostasis, which are the most primary demands for cells under the deprivation or limitation of nutrients and oxygen. Simultaneously, the anaplerotic pathway is promoted to refill the macromolecular biosynthesis for rapid proliferation in some cells. Cells adapt to environmental changes through metabolic remodeling, in order to maintain cellular homeostasis, which is an important stress-protective mechanism that plays a key role in many biological activities (see Figure 3).

### 2.2. Metabolic Reprogramming by Ischemic Preconditioning

**Figure 4:**
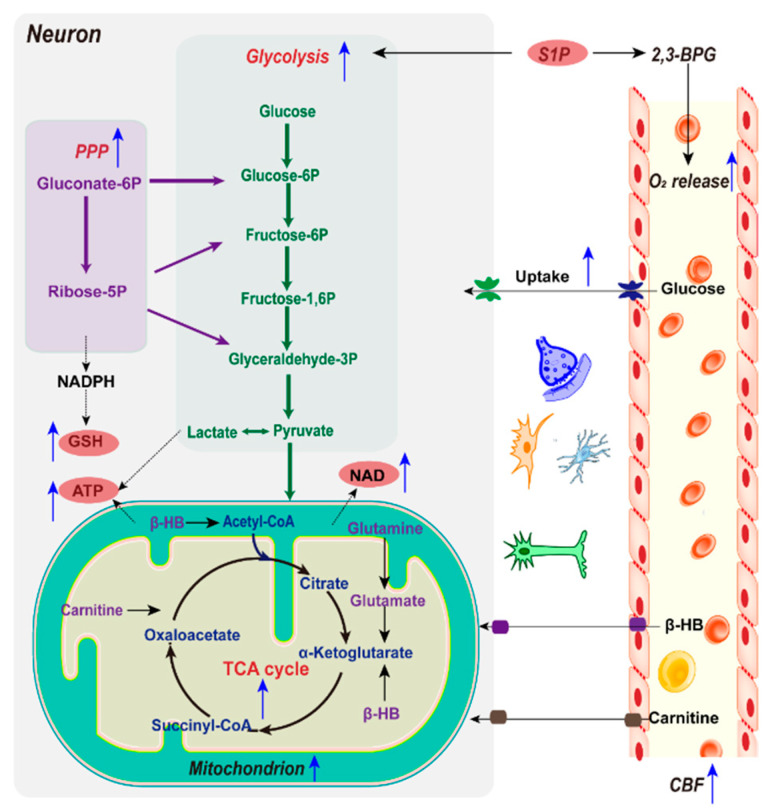
Metabolic reprogramming by ischemic preconditioning (IPC). For blood glucose and oxygen supply, IPC increases regional CBF and regulates the oxygen-delivery ability of erythrocytes through sphingosine 1-phosphate (S1P), in order to maintain glucose and oxygen metabolic consumption. To enhance energy reserves, IPC improves mitochondrial efficiency for cellular energy metabolism, boosts glycolysis, and stockpiles and utilizes alternative energy substrates. Meanwhile, IPC also boosts the PPP, providing an essential redox equivalent for GSH regeneration and enhancing the capacity of antioxidant defense.

Apart from complete reperfusion, IPC is a powerful intervention known for reducing ischemic infarct size. The complex underlying mechanisms responsible for the neuroprotection against IPC remain elusive. Related studies have mainly focused on the processes of humoral and neuronal factors interacting to initiate and transmit signals, in order to increase the cerebral blood flow and protect mitochondria to reduce oxidative stress [73], as well as several key enzymes and regulatory factors, such as AMP-activated protein kinase (AMPK), SIRT1, and SIRT574. Though emerging studies have shown that metabolic reprogramming is especially critical in IPC, the study of metabolic reprogramming conducted by IPC is still in its infancy (Figure 4). The more we understand the underlying metabolic reprogramming mechanisms manipulated by IPC affecting its efficiency and function against ischemic stroke, the more we will be able to experimentally (and, eventually, clinically) utilize the metabolic homeostasis to confer protection against the ischemic insult.

#### 2.2.1. Glucose and Mitochondria

The accumulation of glucose is the primary feature of ischemic stroke, mainly regulated by AMPK, which is a key kinase activated by energy failure which can promote glucose uptake. Research has found that brain ischemia-refusion (I/R) injury can activate AMPK, which is an adaptive response to stress that plays an essential role in maintaining energy homeostasis, while the overactivation of AMPK accentuates hyperglycolysis, which can lead to serious metabolic distress. For ischemic rats, 24 h after IPC treatment, the AMPK levels and glucose levels decreased and ATP increased in the penumbra, indicating that glucose catabolism is upregulated by IPC [18]. However, a controversial finding has been observed in the plasma of ischemic rats and the CSF of humans after IPC: Both of their glucose levels increased significantly [74], indicating the metabolic regulation of IPC may be metabolic compartmentalization. Simultaneously, IPC increases regional CBF, in order to enhance the supply of blood glucose and oxygen to maintain metabolic consumption. Furthermore, IPC treatment also remarkably improves the metabolic disturbances in the TCA cycle during ischemia. IPC has also demonstrated neuroprotective activity through the activation of Nrf2 both in vivo and in vitro, which is a transcription factor that helps to maintain mitochondrial coupling and antioxidant protein expression [75]. This described evidence highlights the capability of IPC in improving mitochondrial efficiency and regulating the reprogramming processes related to mitochondrial function and cellular metabolism.

#### 2.2.2. Glycolysis

Upon ischemic stroke, cerebral glycolysis exhibits an increasing trend. Though glycolysis is advantageous for rapidly producing ATP to meet the high energy demands, hyperglycolysis can aggravate the brain damage caused by lactic acidosis and ROS overproduction [76]. Lactate levels have been shown to decrease 24 h after IPC treatment in MCAO rats, indicating that the glycolytic pathway is downregulated by IPC; meanwhile, the activity of fructose-2,6-biphosphatase 3 (PFKFB3) was inhibited by IPC. PPFKFB3 controls glycolytic flux by synthesizing fructose-2,6-bisphosphate (F2,6BP), a potent allosteric activator of phosphofructokinase 1 (PFK-1), which is a master regulator of glycolysis [18]. Furthermore, the level of glycolytic products of lactate in CSF was found to be decreased following IPC [75]. Altogether, these results imply that subduing postischemic hyperglycolysis and the regulation of brain glucose metabolism play important roles in the neuroprotective aspect of IPC.

#### 2.2.3. NAD^+^/NADH

Preserving pools of NAD^+^ confers neuroprotection after ischemic stress. IPC has been shown to enhance levels of NAD^+^ in the brain [77]. Exogenous application of nicotinamide mononucleotide (NMN), an intermediate of NAD^+^ synthesis, mimics the protective effect of IPC under ischemia and reperfusion injury. An untargeted metabolomics study has revealed that α-hydroxybutyrate (α-HB) stands out as highly significantly upregulated after IPC [78], while previous studies have shown that an elevation in the cytosolic NADH/NAD^+^ ratio would promote α-HB formation. α-HB is a biomarker of the cytosolic NADH/NAD^+^ ratio [79], indicating that IPC can regulate the NADH/NAD^+^ ratio. The regulation of NAD^+^ by IPC is related to NAMPT (nicotinamide phosphoribosyl transferase). Studies have shown that IPC upregulates NAMPT protein, and the protective effect of IPC against ischemia (30 min) and reperfusion (24 h) was attenuated in NAMPT knockdown mice, suggesting that NAMPT is essential in mediating the protective effect of IPC [80]. NAMPT levels were enhanced by protein kinase C (PKC) in an AMPK-dependent manner, which was required for increased mitochondrial NAMPT after IPC [77].

#### 2.2.4. NADPH and GSH

As the main product of the oxidative PPP (oxPPP), NADPH provides the essential redox equivalent for GSH regeneration, enhancing the antioxidant defense capacity. As the brain NADPH level decreases during ischemia, boosting the PPP activity may serve as a potential neuroprotective strategy for the regulation of the cellular redox environment [81]. Previous evidence has revealed that IPC diverts excess glucose to oxPPP. Meanwhile, glucose-6-phosphate dehydrogenase (G6PD) activity was notably increased by IPC in both normal and ischemic brains, which is the key rate-limiting enzyme responsible for regulating the flux of glucose into the PPP. Elevated PPP flux enhances the reducing power, in the form of NADPH and GSH. Additionally, GSH is synthesized from glutamate, cysteine, and glycine. Glutamine importantly regulates this process by providing glutamate and promoting cystine uptake [82]. Significantly lower levels of glutamine and glycine in CSF following IPC have been observed [75], indicating that the replenishment of GSH is accelerated, eventually imposing reductive stress in the ischemic brain tissue.

#### 2.2.5. Alternative Energy Substrates

Accumulating evidence has suggested that IPC regulates the cerebral metabolism by providing alternative energy substrates, which partly reduce the dependence of the brain on a continuous supply of glucose, therefore improving the brain’s resistance to ischemia. ➀ Ketone: Notably, the brain and plasma β-hydroxybutyrate (β-HB) levels both increase under IPC stimulation, indicating that the brain can increase ketone body oxidation to replenish its energy supply. Efficient ketone metabolism generates relatively abundant energy, which may prevent activation of the hyperglycolytic pathway under oxygen and glucose deprivation [27]. ➁ Phosphocreatine (PCr): In addition, the protective effect of IPC on metabolic recovery has been demonstrated by a notably increased level of creatine, observed both in rat plasma and human CSF [27,75], suggesting an improvement in the neuroenergetic status. Creatine generally occurs as phosphocreatine (PCr), which is a storage form of high-energy phosphate and a shuttle for the transfer of high-energy phosphate from mitochondria to the cytosol. However, the importance of PCr in energy homeostasis is underestimated by the fact that the total creatine pool (as creatine and PCr) in the brain is at least three-fold larger than the adenosine nucleotide pool (consisting of AMP, ADP, and ATP). ➂ L-Carnitine: The level of lysine in human CSF increases following IPC [75]. It is well-known that lysine, being an energy-providing amino acid, is necessary for the biosynthesis of L-carnitine. L-carnitine is the only transporter of fatty acids across the mitochondrial membrane, to be metabolized with the generation of energy, indicating an energetic compensatory mechanism by IPC for neuronal survival. ➃ Lactate: Previous research has suggested that astrocytes play a pivotal role in the induction of ischemic tolerance [83], during which lactate is extremely crucial. The study conducted cerebral ischemia and IPC in cultured rodent astrocytes and neurons, revealed that neurons incubated with IPC-treated astrocytes were significantly protected against lethal ischemic injury. Meanwhile, IPC-treated astrocytes significantly enhanced lactate secretion into the extracellular media. Eventually, exogenous lactate administration can significantly increase cell survival in neuronal cultures against lethal oxygen glucose deprivation (OGD) [84]. Fluorodeoxyglucose–positron emission tomography (FDG-PET) has demonstrated the preferential utilization of lactate over glucose to fuel neurons, when both were available [85]. This process is named the astrocyte–neuron lactate shuttle (ANLS). It has been implied that the neuroprotective ability of IPC may be related with the promotion of ANLS, where lactate serves as a potential agent to protect neurons against lethal ischemic injury.

#### 2.2.6. Sphingosine 1-Phosphate and O_2_ Delivery

Oxygen is a crucial substrate in metabolism. Erythrocytes are the only cell type responsible for delivering oxygen. To the best of our knowledge, no study has assessed whether IPC affects the oxygen delivery ability of erythrocytes. Sphingosine 1-phosphate (S1P) is a bioactive signaling lipid highly enriched in mature erythrocytes. Previous research has revealed that S1P is an important endogenous protectant against ischemia, where the increased release of S1P from myocytes in response to IPC has been observed [86]. Recently, Yang X. et al. revealed that S1P promoted erythrocyte glycolysis and oxygen release for adaptation to hypoxia. They showed erythrocyte S1P levels rapidly increased in 21 healthy lowland volunteers at 5260 m altitude, with a concurrent elevation of hemoglobin and oxygen release capacity. Mouse genetic studies have shown that S1P protects against tissue hypoxia by inducing O_2_ release. Mechanistically, S1P enhances the release of membrane-bound glycolytic enzymes to the cytosol, which promotes glycolysis and, thus, increases the production of 2,3-bisphosphoglycerate (2,3-BPG). 2,3-BPG is an erythrocyte-specific glycolytic intermediate that facilitates O_2_ release [71]; concurrently, hypoxia promotes renal damage and progression of chronic kidney disease (CKD). There is also a beneficial role of erythrocyte S1P in hypertensive CKD, where S1P also induces 2,3-BPG production and oxygen delivery [72]. Together, these findings reveal the biological activity of S1P in erythrocyte oxygen delivery, indicating that IPC may enhance erythrocyte oxygen delivery through S1P, thereby enhancing cerebral metabolism to defend against ischemic stress.

### 2.3. Spatiotemporal Variation in IPC Metabolomic Reprogramming

It should be noted that, due to different synaptic activities, loop connectivity, and functional domains, heterogeneity exists among the spatial distribution of endogenous metabolites; this distribution characteristic has only been noticed in recent years. In 2014, Gary used laser desorption–ionization mass (LDI/MS) spectrometry to create maps of the spatial distributions of glutamine, DHA, and other metabolites across the brain and within each sub-region [87]. In 2020, Per E. Andrén et al. utilized MALDI-MSI to observe the intracerebral distribution of neurotransmitters in Parkinsonian rats, primates, and human patients [88]. Both studies revealed that the metabolites have inhomogeneous distributions in the brain, with high levels of spatial specificity. This feature determines that the metabolic homeostasis of neurons is related to their brain micro-environment, which may provide different substrates to fuel the neurons. Furthermore, such heterogeneous distribution of metabolic substrates may be exploited by different brain regions, in order to regulate their cellular metabolic homeostasis during mitochondrial dysfunction. The research by Polyzos et al. confirmed this: In a Huntington model, they discovered region-specific metabolic reprogramming of astrocytes, which directly induced neuronal susceptibility. The vulnerable striatum is enriched in fatty acids, which the mitochondria reprogram to be metabolized as an energy source, but at the cost of ROS accumulation and induced damage. Meanwhile, the cerebellum is replete with amino acids, which are precursors for glucose regeneration through the pentose–phosphate shunt or gluconeogenesis pathways. ROS is not elevated and, so, this region sustains little damage [89]. Considering these results, we may be able to predict the spatial properties of ischemic stroke metabolic disorders and IPC-mediated metabolic remodeling; however, there is still a lack of relevant research.

Cellular metabolism is a flexible network to meet homeostasis demands in real-time. This is typical in cancer progression, as primary tumor cells rely on anabolic metabolism to maintain cell proliferation; then, when they enter the circulation, their survival requirement shifts to produce NADPH and GSH, in order to counteract oxidative stress. Interestingly, IPC has exhibited significant time-window effects in both basic and clinical research, where two phases have been observed. In the early phase, tissues benefit within minutes of IPC intervention, lasting for 2–3 h, while the late phase occurs at 12–24 h and lasts for 2–3 days. A previous study has revealed that, once IPC is conducted, autacoids stimulate a number of signaling pathways that convey a protective signal to the mitochondria, where signaling ROS are generated and activate protein kinases to provide the “memory”; this process can last up to 2–3 h. Meanwhile, evidence demonstrated the neuroprotection of IPC may depend on the activation of adenosine A1 receptors [90]. Noteworthy, at the early ischemia-reperfusion (I/R) phase, the impaired mitochondrial function was attenuated by IPC and mediated by adenosine A1 receptors [91,92]. In delayed IPC, those protein kinases activate transcription factors, which facilitate the synthesis of distal mediators, such as cyclooxygenase-2 (COX-2) and heat shock protein (Hsp72), to induce the protective effect 12–24 h after IPC. An increasing number of studies have shown the time-dependent metabolic changes during IPC or the acute-to-chronic post-stroke phase. A self-controlled interventional study measured dynamic cerebral autoregulation (dCA) and blood biomarkers at seven time points in healthy participants who had conducted IPC, and found that dCA was significantly increased at 6 h and was sustained for at least 24 h after IPC, while two neuroprotective factors and four inflammation-related biomarkers were significantly elevated, compared with their baseline levels. These studies have indicated the time-specificity of IPC; however, the dynamic change of metabolic reprogramming induced by IPC is still unclear. Another critical concern is the proper time window for IPC metabolic reprogramming in sustaining the neuroprotection effects for the forthcoming ischemia stroke. Previous studies reported that when MCAO is applied during the early IPC phase, the brain is ischemia-tolerant. In subsequent hours after IPC, the brain regresses to its naïve state. If ischemic stress is applied during this phase, the neuroprotection effects of IPC vanish. Whereas in the delayed or the second protect phase of IPC, the brain is again ischemia-tolerant [93]. Together, effective IPC metabolic reprogramming may happen and be assumed to sustain during the early and late phases of IPC. Detailed metabolomics data verification with higher time and tissue-specific resolution will be needed in the future.

### 2.4. Influence of Aging on IPC Metabolic Reprogramming

Ischemic stroke occurs most frequently in individuals aged ≥65 years. Coincidentally, a clinical study has implicated the effectiveness of IPC in preventing the progression of white matter hyperintensities (WMHs) and in ameliorating cognitive impairment of very elderly patients (83.5 ± 2.3 year) with ICAS [94]. Studies also clearly demonstrate that IPC is quite effective in aged animals: in aged gerbils, IPC provided substantial neuroprotection (>80%) in CA1 neurons ten days after ischemia compared with 6% in ischemic gerbils [95]. Though the information about IPC-mediated metabolic reprogramming in older adults is scant, these promising findings drive the hypothesis that IPC-mediated metabolomic reprogramming may have a subtle susceptibility to aging. Consistent with IPC may effectively reprogram mitochondrial energy metabolism and benefit stroke. It was shown that free and protein-bound NADH differs regarding lifetime. The latter form is informative in energy metabolism than free NADH. In addition, age-related alterations in TCA cycle enzyme activities will likely contribute to the decline of mitochondrial bioenergetics [96]. These preliminary results prompt the research on pathway-specifics alterations in metabolic reprogramming with aging during the stroke and IPC, which will be crucial for precision intervention for individuals of different ages, especially for patients with advanced age and a higher ischemic stroke incidence rate.

### 2.5. Astrocyte-Neuron Interactions in IPC Metabolic Reprogramming

Since astrocytes play an integral role in inducing ischemic tolerance [97], the traditional view of astrocytes as passive supporters of neurons is revised, and the survival of neurons tightly associated with astrocytes is recognized. However, the details of how metabolite coupling between astrocyte and neurons in stroke are still not clear, and the understanding of metabolic pathway regulation during IPC metabolic reprogramming is just beginning. In the mammalian brain, neurons are outnumbered 10:1 by astrocytes in most regions. Astrocytes state in a particular position to both sense neuronal signaling and capture glucose directly from the capillary and permit them to govern astrocyte-neurons cooperation. Notably, neurons and astrocytes preferentially use quite different metabolic pathways in physiological conditions [98]. As mentioned before, astrocytes play an essential role in the re-flux of glucose into neurons for energy production and utilization. The glucose taken up by astrocytes may have one of two primary fates: it may be converted to lactate through astrocytic glycolysis or converted via glycogenesis to glycogen storage. Importantly, in adult neurons, to meet the higher energy requirements, neurons sustain a high rate of oxidative metabolism compared to astrocytes, by which aerobic glycolysis results in the generation of pyruvate, not lactate. As the storage form of glucose, the polymer glycogen is entirely located in astrocytes, and the glycogen metabolism rarely occurs in neurons [99]. Remarkably, specific neurotransmitters and neuromodulators could dictate astrocytes glycogenolysis. The astrocytic glycolysis is also stimulated by neuronal activation, giving neurons the capacity of tight control over astrocyte metabolism. One study showed that neurons made specific metabolic adaptations following IPC (transient OGD) with the regulation of oxygen utilization and lactate production [100]. Consistently, a study showed that neurons benefited from the co-incubated astrocytes, enhancing lactate secretion induced by IPC in astrocytes. Collectively, these findings suggest IPC could specify metabolic reprogramming in neurons and astrocytes and contribute to functional homeostasis. However, due to the structural complexity and their specific physiological functions and metabolic patterns, the conclusive details on whether the dynamic metabolic reprogramming behavior accompanied with astrocyte-neuron interaction is induced by ischemia or IPC are still lacking. Advance in this active research field will stimulate a promising new direction in precision intervention and drug target discovery for ischemic stroke.

## 3. Conclusions and Perspectives

Above all, metabolism is essential for life activities. Mounting evidence has shown that brain metabolic plasticity and IPC metabolic reprogramming are crucial for ischemic defense, typically through maintaining cellular energy and redox homeostasis. However, the complex connection between the neuroprotective function of IPC and cerebral metabolic reprogramming is still an exciting area of investigation, especially with respect to their spatiotemporal variation in consideration of the brain metabolic compartmentalization and time dependence. Furthermore, as IPC not only can salvage the stroke patient at the acute period, but can also provide effective solutions for stroke rehabilitation during the chronic period, determination of the underlying metabolic regulation mechanism, which is still unclear, should be actively pursued. Furthermore, except for NADPH and GSH, whether there exist some other mechanisms induced by IPC to maintain the redox homeostasis under ischemia is not yet known; especially considering ferroptosis, which has been implicated in the pathological cell death associated with neurodegenerative diseases (i.e., Alzheimer’s, Huntington’s, and Parkinson’s diseases). Intriguing, the protective effect of IPC can be mimicked pharmacologically. Several studies also showed that transient ischemic attack (TIA) may produce IPC effect in people who have a subsequent stroke [101,102]. Preservation of the IPC phenotype implicated a unifying endogenous mechanism, possibly involving energy and redox homeostasis maintenance. Likewise, as the most difficult challenge in ischemic stroke is energy failure, whether some other new energetic substrates are mobilized by IPC (e.g., fructose), in addition to the glucose and common alternative energy substrates, should be determined. In-depth research considering these open questions will be valuable for exploring the mechanisms of IPC. In short, understanding the mechanism of metabolic reprogramming is expected to be greatly beneficial for our understanding of ischemic stroke treatment and for the standardized application of IPC.

## Figures and Tables

**Table 1:** Metabolic syndrome (MetS) increases stroke incidence.

Study	Main Results	Reference
Cohort study of 5398 adults aged 35 years or older followed for 10 years	Stroke incidence rates for those with and without MetS were 2.6% and 1.1%, respectively.	[58]
Cohort study of 1361 outpatients	40.2% ischemic stroke individuals were diagnosed with MetS.	[59]
Cross-sectional study of 840 patients	MetS patients had a 3.542-fold increased odds ratio (OR) for cognitive impairment.	[60]
Trial of 2860 patients and followed them for 3.5 years	When ischemic stroke occurred, patients who had a target LDL cholesterol level of 90–110 mg per deciliter had a higher risk of subsequent cardiovascular events than those who had a target range of less than 70 mg per deciliter.	[61]
U.S. National Health and Nutrition Examination Surveys of 12,502 adults during 1999–2010	MetS prevalence was 61.2% in stroke survivors.	[62]

## Data Availability

Not applicable.
